# Mapping Care Partner-Reported Distressing Behaviors to Speech-Language Pathology Interventions

**DOI:** 10.1093/geroni/igaf122.175

**Published:** 2025-12-31

**Authors:** Aimee Mooney, Allison Lindauer, Christina Zonker, Heather Franklin

**Affiliations:** Oregon Health & Science University, Portland, Oregon, United States; Oregon Health & Science University, Portland, Oregon, United States; Oregon Health & Science University, Portland, Oregon, United States; Oregon Health & Science University, Portland, Oregon, United States

## Abstract

**Purpose:**

Speech and Language Pathologists play a central role in the assessment, diagnosis, and treatment of persons with dementia (PWD) and their caregivers. The World Health Organization (WHO) has directed rehabilitation as a core component of dementia care. Dementia caregivers commonly relate distressing behaviors, e.g. “withdraws during family functions” and “repetitive questions; interrupting”. This study maps distressing behaviors reported by dementia caregivers to Non-Pharmacological Treatments (NPTs), inclusive of Cognitive Rehabilitation, Expressive-Receptive Language Therapy and Swallowing.

**Method:**

Distressing behaviors were identified by 176 family caregivers as part of a larger psychoeducational intervention project: Tele-STELLA, (Support via Technology: Living and Learning with Advancing dementia, NIA R01AG067546). Definitions, treatment targets and treatment components were condensed into a final catalog of seven NPTs; coding rules were established. Two researchers mapped an initial sampling of 98 behaviors onto the NPTs, achieving substantial inter-rater reliability, with an agreement of 79% and a Cohen’s Kappa of 0.68 [95% CI (0.56, 0.80)].

**Results:**

73% of the behaviors were categorized to the NPTs of Cognitive Rehabilitation, (43), Expressive/Receptive Language Therapy/Swallowing (16) and Psychological Management (13); 2% of behaviors were coded as ‘No NPT.

**Conclusions:**

By categorizing the caregiver-generated distressing behaviors into applicable NPTs, we highlight the need for referral to Speech and Language Therapy in dementia care. This analysis suggests many dementia caregiver concerns map neatly to SLT interventions focusing on cognitive, behavioral, communication/language and swallowing challenges. This provides practical information for gerontologists and other healthcare professionals when considering referrals to optimize function and reduce disability in dementia care.

